# Effect of population breast screening on breast cancer mortality up to 2005 in England and Wales: an individual-level cohort study

**DOI:** 10.1038/bjc.2016.415

**Published:** 2016-12-08

**Authors:** Louise E Johns, Derek A Coleman, Anthony J Swerdlow, Susan M Moss

**Affiliations:** 1 Division of Genetics and Epidemiology, The Institute of Cancer Research, London SM2 5NG, UK; 2 Division of Breast Cancer Research, The Institute of Cancer Research, London SW3 6JB, UK; 3 Centre for Cancer Prevention, Wolfson Institute of Preventative Medicine, Queen Mary University of London, Charterhouse Square, London EC1M 6BQ, UK

**Keywords:** breast cancer, mortality, population screening, evaluation, incidence-based mortality, individual-level

## Abstract

**Background:**

Population breast screening has been implemented in the UK for over 25 years, but the size of benefit attributable to such programmes remains controversial. We have conducted the first individual-based cohort evaluation of population breast screening in the UK, to estimate the impact of the NHS breast screening programme (NHSBSP) on breast cancer mortality.

**Methods:**

We followed 988 090 women aged 49–64 years in 1991 resident in England and Wales, who because of the staggered implementation of the NHSBSP, included both invited subjects and an uninvited control group. Individual-level breast screening histories were linked to individual-level mortality and breast cancer incidence data from national registers. Risk of death from breast cancer was investigated by incidence-based mortality analyses in relation to intention to screen and first round attendance. Overdiagnosis of breast cancer following a single screening round was also investigated.

**Results:**

Invitation to NHSBSP screening was associated with a reduction in breast cancer mortality in 1991–2005 of 21% (RR=0.79, 95% CI: 0.73–0.84, *P*<0·001) after adjustment for age, socioeconomic status and lead-time. Breast cancer deaths among first invitation attenders were 46% lower than among non-attenders (RR=0.54, 95% CI: 0.51–0·57, *P*<0.001) and 32% lower following adjustment for age, socioeconomic status and self-selection bias (RR=0.68, 95% CI: 0.63–0·73, *P*<0.001). There was little evidence of overdiagnosis associated with invitation to first screen.

**Conclusions:**

The results indicate a substantial, statistically significant reduction in breast cancer mortality between 1991 and 2005 associated with NHSBSP activity. This is important in public health terms.

Population breast screening was introduced in a number of countries from the late 1980s after randomised controlled trials reported that mammographic screening could reduce breast cancer mortality by an average of 23% in invited women aged 50–69 years (Lauby-Secretan *et al*, 2015). Debate, however, continues about the relative benefits and disadvantages of such screening programmes (Paci *et al*, 2014; Bleyer *et al*, 2016; Jorgensen and Gotzsche, 2016). This, coupled with ongoing improvements in breast cancer treatment, has led to questions about the value of population screening in reducing breast cancer mortality, and highlighted the need to evaluate the effectiveness of existing population breast screening programmes.

With the exception of Finland, population-based breast screening has been introduced without intrinsic provision for evaluation, making identification of an uninvited comparison population difficult. The use of individual-level data to take into account the screening history of each woman and to identify breast cancers diagnosed before invitation has been strongly recommended (Broeders *et al*, 2012; Weedon-Fekjaer *et al*, 2014). However, such data are not readily available and relatively few evaluation studies have used individual screening and outcome data.

The UK NHS Breast Screening Programme (NHSBSP) is one of the largest nationally organised programmes in the world, currently inviting nearly three million women each year (Health and Social Care Information Centre, 2016). The NHSBSP was introduced in 1988 inviting women aged 50–64 years every 3 years. Implementation of the NHSBSP was gradual, with the first screening round not completed until 1995.

To evaluate the impact of the NHSBSP in England and Wales on breast cancer mortality we conducted a retrospective cohort analysis using individual-level screening exposure and mortality outcome data. The staggered implementation period of the programme was used to provide an uninvited control group. This paper presents an analysis of the impact of NHSBSP activity on breast cancer mortality between 1991 and 2005.

## Materials and methods

We describe our methods briefly below, with additional details provided in Supplementary Material.

### Subjects and data acquisition

The study area covered around one third of England and the whole of Wales (Supplementary Figure A), designed to include the earliest and latest areas to begin NHS screening. The cohort were women aged 49–64 years, resident in the study area and free from breast cancer on 1 January 1991. Breast screening histories were extracted from screening call/recall databases in the study area. Personal details were augmented using data from the NHS Strategic Tracing Service (NSTS; now the Demographics Batch Service, part of the NHS Care Records Service) to aid linkage between different sources of the data. Dates of death were obtained from screening call/recall databases, the NSTS, and the Office for National Statistics (ONS). Data on underlying cause of death were collected from ONS and breast cancer deaths were those for which breast cancer was coded as the underlying cause. Data on incidence of breast cancer, including *in situ* disease, were collected from the national cancer registration system. Socioeconomic status (SES) was estimated based on postcode of residence at study entry using the Townsend Index (Phillimore *et al*, 1994) based on data from the 1991 census (Supplementary Materials 1, 2 and 3).

**Figure 1:**
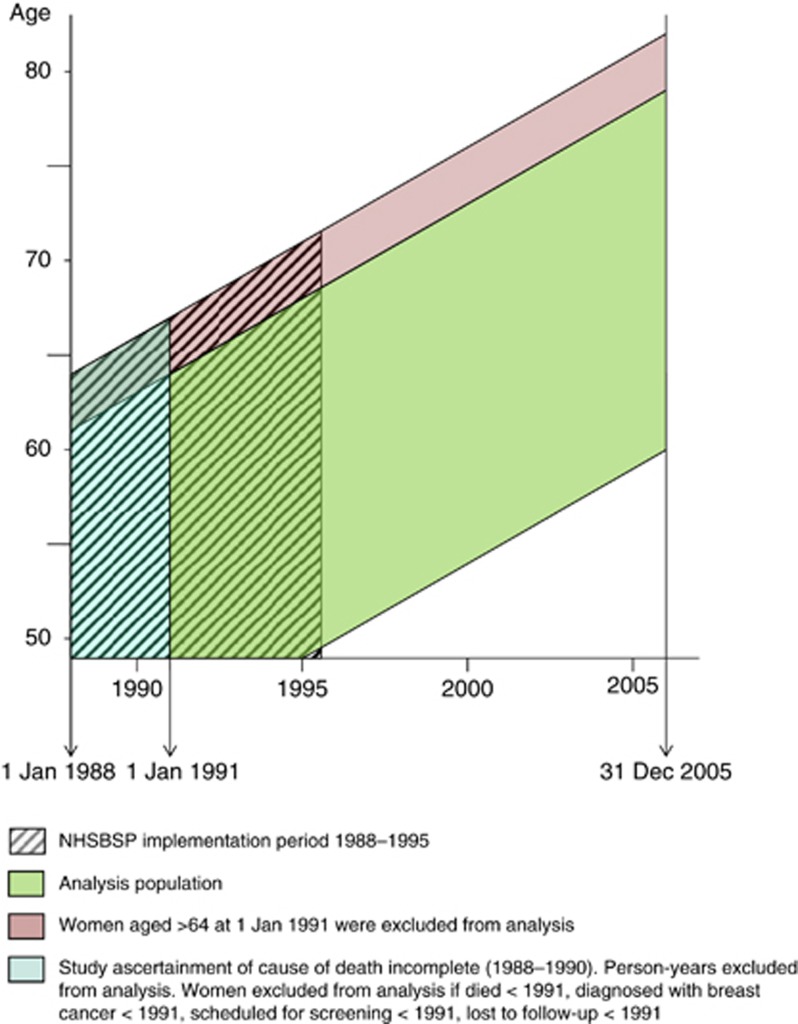
Study population and women available for analysis.

Data collection from ONS was accomplished by individual-level electronic record linkage to the screening data. The NHSBSP in England and Wales started in 1988, but data on cause of death collected by linkage for 1988–1990 were incomplete and therefore analyses for this study started in 1991 (Supplementary Material 5). The analysis population for this study is shown in the lexis diagram, Figure 1. Data linkage was complex and time-consuming and was accomplished to the end of 2005. Follow-up ended at 31st December 2005.

The study protocol was approved by the Department of Health for England, ethics approval was obtained from the South East MREC (MREC 02/01/64) and exemption from individual informed consent was approved by the then Patient Information Advisory Group (PIAG 3-07(g)/2002).

### Analyses

Women entered the study on 1 January 1991 and exited at date of death, date lost to follow-up or 31 December 2005, whichever was earliest (Supplementary Material 4). Primary analysis was conducted on an ‘intention-to-screen' basis (i.e., ‘exposed' women were those invited for screening, not just those who attended). In the NHSBSP, women were scheduled for invitation on the basis of demographic information and their further eligibility was checked by their GP practice before being invited. This process of checking suitability for screening with GPs on the basis of health status (a process that has now ceased) meant that ill-health could result in a scheduled woman not being invited at that episode. Under these circumstances, use of invitation as the measure of exposure in an intention to screen analysis could have led to an estimate that was biased in favour of screening because of a healthy invitee effect (Supplementary Material 6). To avoid healthy invitee bias, we therefore used scheduling for invitation as the measure of exposure in our intention-to-screen analyses (referred to below simply as ‘invitation'). To reflect the potential for women to move between exposure groups over time, the intention-to-screen analysis of mortality used incidence-based mortality (IBM), in which deaths from breast cancer were assigned to the woman's exposure group at diagnosis (Njor *et al*, 2012). This excludes deaths in breast cancer diagnosed before the start of the study period, and ensures that a woman who dies after invitation to breast screening from a breast cancer diagnosed before invitation is counted as a death in the unexposed group. The analysis was designed to ensure that women with breast cancer had the same length of follow-up in both the exposed and unexposed groups. The 15-year period 1991–2005 was partitioned into observation periods that were of equal length in both the invited and uninvited groups. Each observation period included an initial period of 2 years during which breast cancer cases and person-years were accrued, and extended up to 9 years, measured from the start of the observation period, to follow-up for death in any of these breast cancers. As most women were invited for screening relatively soon after the start of the study, this allowed for one observation period in the unexposed group and up to three observation periods in the exposed group. The strict partitioning of time in this analysis resulted in the inclusion of screening activity up to year 2000. Full details of the IBM analysis methodology are described in the Supplementary Material 7.

Lead-time bias consequent on screening advancing the date of diagnosis can bias results against a positive effect of screening in IBM analysis by including breast cancer deaths in women who would otherwise have been diagnosed beyond the accrual period (Njor *et al*, 2012). Intention-to-screen analyses were adjusted for this bias (Supplementary Material 8) assuming a lead-time of 3 years for screen-detected cases (Supplementary Material 3), based on published estimates of lead-time (Weedon-Fekjaer *et al*, 2005; Svendsen *et al*, 2006). Analyses were repeated using lead-time estimates of one, 5 and 7 years.

A conventional mortality analysis of intention to screen was also conducted in which deaths were allocated to exposure groups at the date of death and breast cancer mortality in women living in the earliest and latest areas to start screening was compared. We designated as early-starting postcode districts those where ⩾95% of women were first invited before December 1991 and late-starting areas where ⩾95% of women were first invited after July 1993. Follow-up continued until the end of 2005.

We conducted an analysis based on screening attendance, dividing women at entry according to whether or not they had attended within 6 months of their first screening invitation. In the NHSBSP, if a woman does not attend within 6 months of her invitation, the episode is closed. The limit of 6 months therefore ensures that in our analysis any attendance relates directly to the correct invitation. In this analysis, only women who had been sent an invitation were included. Estimates were adjusted to take account of the increased mortality risk in women who do not accept screening (self-selection bias; Duffy and Cuzick, 2002), using information on uninvited women from the cohort to derive a population-specific correction factor (Supplementary Material 9).

To investigate overdiagnosis of breast cancer due to screening, a cumulative incidence analysis (Biesheuvel *et al*, 2007) of invasive and *in situ* breast cancers diagnosed in women with the earliest birth years in the cohort (1927–1929) was conducted. Some of these women, due to their age and the staggered introduction of screening, were never invited by the NHSBSP. Those who were invited would have had only a single invitation (women were not invited above age 64 years at that time), giving at least 12 years of follow-up for lead-time to dissipate so that any remaining excess incidence in the invited group can be attributed to screening. Excess incidence has been expressed both as a proportion of observed incidence in uninvited women and of that in invited women, methods A and B advocated by the Independent UK Panel on Breast Cancer Screening (2012); Supplementary Material 10.

Standardised mortality rates (SMRs; i.e., mortality rates in the cohort compared with national rates), adjusted by age and calendar year were used to compare non-breast cancer mortality between exposure groups (Supplementary Material 11).

The number of women needed to be invited to save one breast cancer death was calculated as the reciprocal of the number of lives saved per woman invited (Supplementary Material 12). The number needed to be screened to save one breast cancer death was calculated according to the method of Richardson, (2001) (Supplementary Material 13).

Poisson regression, conducted in STATA V10·0 (StataCorp, College Station, TX, USA), was used to calculate rate ratios and associated 95% confidence intervals and *P*-values. Age and socioeconomic status were included as covariates in the model.

## Results

Data on a total of 1 426 379 women aged 49–64 years on 1 January 1991 were extracted from 28 screening call/recall databases. Of these, we excluded from analyses women who were not traced at NSTS (14 157), women with breast cancer diagnosed before 1 January 1991 (28 870) and women invited before 1 January 1991 (395 262). This resulted in an analysis population of 988 090 women.

Between 1 January 1991 and 31 December 2005, there were 41 120 cases of breast cancer diagnosed and 146 539 deaths in the cohort, including 8002 deaths from breast cancer. Linkage failed to produce an underlying cause of death for 2% of deaths (3032/146 539). To determine whether any of the missing causes of death were due to breast cancer, all women whom we knew from cancer registration had been diagnosed with breast cancer but whose cause of death was not known (*n*=101) were flagged at the NHS central register to determine an underlying cause. None was found to have died of breast cancer. A total of 39 134 women (4%) were lost to follow-up for reasons other than death before 31 December 2005, and a further 8014 who left the study area before being scheduled for NHSBSP invitation were lost to follow-up in the IBM analysis.

### Intention-to-screen analyses

#### Breast cancer incidence-based mortality in invited compared with uninvited women

Breast cancer mortality was 17% lower in women invited for screening than in uninvited women (RR=0.83, 95% CI: 0.78–0.89, *P*<0.001; Table 1), and the estimate was similar following adjustment for age and SES (RR=0.82, 95% CI: 0.76–0.88, *P*<0.001). After adjusting for lead-time bias of 3 years, the mortality reduction increased to 21% (RR=0.79, 95% CI: 0.73–0.84, *P*<0.001). The absolute difference was 0.31 per 1000 person-years and the number needed to be invited to save one death from breast cancer was 1436 (based on inviting women for 2 years and 9 years of follow-up). Non-breast cancer SMRs in the invited and uninvited groups were 0.96 (0.95–0.97) and 0.98 (0.96–1.00), respectively, SMR ratio 0.98.

#### Breast cancer mortality in early- compared with late-starting screening areas

Early- and late-starting areas had populations of 49 713 and 52 949 study women, respectively. Mean follow-up in this analysis was 13.7 years. Adjusted for age and SES, breast cancer mortality in the early-starting areas was 18% lower than in the late-starting areas (RR=0.82, 95% CI: 0.71–0·94, *P*=0.004; Table 1).

### Breast cancer mortality in screening attenders compared with non-attenders

A total of 790 946 women were invited to their first screen between ages 49 and 64 years, and 587 809 (74%) attended within 6 months. The breast cancer mortality reduction in women who attended their first screen compared with those who did not attend was 46% (RR=0·54, 95% CI: 0.51–0.57, *P*<0.001) and this estimate was unaffected when adjusted for age and SES (Table 2). The absolute difference was 0.50 per 1000 person-years and the number needed to be screened in order to save one death from breast cancer was 1020 (where screened women attended a first screen within 6 months of invitation and attended on average 2.8 screens over a mean 12.3 years follow-up). After adjustment for self-selection bias, using the population-specific correction factor of 1.19, the mortality reduction was 32% (RR=0·68, 95% CI: 0.63–0.73, *P*<0.001).

### Analyses of overdiagnosis of breast cancer

A total of 162 502 women aged 62–64 years at entry contributed 2 033 325 person-years of follow-up during which 6108 breast cancers were diagnosed. Lead-time effects were apparent when follow-up was censored at the end of 1995 or the end of 2000, but had disappeared by 2005. At the end of follow-up, the cumulative incidence rates of breast cancer adjusted for SES in the invited and uninvited groups were 3.01 (95% CI: 2.89–3.13) and 3.00 (95% CI: 2.90–3.10) cases per 1000 person-years, respectively (*P*=0.90 for a difference between groups). This equates to 0.3% overdiagnosis after one invitation and 12 years of follow-up as a percentage of the observed incidence in either invited or uninvited women.

## Discussion

This is the first cohort study using individual-level data to evaluate NHS breast screening in the UK, and worldwide it is one of the largest IBM evaluations of population breast screening that has been conducted. Our findings suggest that breast cancer mortality was 21% lower in women invited by the NHSBSP between 1991 and 2000 compared with women of the same age who were not invited. Among women invited for their first screen, breast cancer mortality was 32% lower in attenders than non-attenders, adjusted for self-selection bias.

Previously published studies specifically reporting the impact of the UK breast screening programme on breast cancer mortality have either relied on modelling aggregated data (Blanks *et al*, 2000; Duffy *et al*, 2010) or have used a case-control approach based on individual-level data (Fielder *et al*, 2004; Allgood *et al*, 2008; Massat *et al*, 2016).

Our results are similar to those from a recent review and meta-analysis of the impact of mammographic screening on breast cancer mortality in Europe published in 2012 for the EUROSCREEN Working Group (Broeders *et al*, 2012). The reviewers identified seven eligible IBM studies, where mortality rates were calculated on the basis of breast cancer deaths occurring in women with breast cancer diagnosed after their first invitation to screening. The reported pooled breast cancer mortality reduction was 25% (RR 0.75, 95% CI: 0.69–0.81) among invited women and 38% among those screened (RR 0.62, 95% CI: 0.56–0.69).

Since this European review, two additional large IBM evaluations of organised breast screening in Norway and Finland have been published (Weedon-Fekjaer *et al*, 2014; Parvinen *et al*, 2015). These studies reported reductions in death from breast cancer of between 25 and 28% associated with invitation to screening. Our UK evaluation is very similar in design to the study in Norway, where screening was implemented gradually between 1995 and 2005. The Norwegian study found a 28% reduction in breast cancer mortality among women invited (RR=0.72, 95% CI: 0.64–0.79) and a 37% reduction associated with screening attendance (Weedon-Fekjaer *et al*, 2014). The greater magnitude of mortality reduction in Norway compared with our UK study might be accounted for by a more recent screening period employing contemporary screening practice, a shorter screening interval (2 years in Norway, 3 years in the UK) and the use of two-view mammography at all screens throughout the Norwegian evaluation period compared with two-views at the first screen only in most of the NHSBSP during the study period.

### Analysis of early *vs* late start of screening

The comparison of mortality in early- and late-starting screening areas showed a relative reduction in breast cancer mortality of 18% in early-starting areas. This is likely to be due to women in the early-starting areas receiving screening over a longer period of time. Although this was a logical evaluation approach, it was subject to dilution by subsequent screening in late-starting areas. Nevertheless, the observed breast cancer mortality reduction of 18% was consistent with the 21% reduction estimated by the more rigorous intention-to-screen IBM approach, albeit with wider confidence intervals.

### Overdiagnosis

As a consequence of breast screening, some early-stage tumours are diagnosed which would never progress to become clinically apparent during a woman's lifetime. This represents overdiagnosis. Whilst our study was primarily designed to estimate the impact of NHS breast screening on breast cancer mortality, the oldest birth cohorts offered an opportunity to investigate overdiagnosis of breast cancer in relation to screening activity. By 2005, lead-time effects in the invited group had dissipated, so that unlike previously published estimates of overdiagnosis in the NHSBSP (Wilson and Evans, 2006; Duffy *et al*, 2010; Jorgensen and Gotzsche, 2010), our analysis indicated only 0.3% overdiagnosis associated with invitation. This increased to 0.5% if expressed as a percentage of cases diagnosed in the first 7 years. Our finding of so little overdiagnosis may be due to our analysis being based predominantly on one invitation per woman, whereas previously published estimates of around 10–15% overdiagnosis in the NHSBSP and elsewhere are based on screening histories including multiple attendances per woman (Duffy and Parmar, 2013; Michalopoulos and Duffy, 2016). A cohort study in Italy used the cumulative incidence method to investigate overdiagnosis in women aged 60–69 years at entry in the first round of the Florentine screening program. After 5–14 years follow-up, they found 5% overdiagnosis of invasive breast cancer alone and 10% overdiagnosis of invasive plus *in situ* breast cancer (1.10 (0.98–1.23); Puliti *et al*, 2012). Our estimate of overdiagnosis increases to 5% if follow-up is restricted to 7 years; however, this is less than the follow-up recommended (Independent UK Panel on Breast Cancer Screening, 2012), and the risk of overestimating overdiagnosis if follow-up is too short has been demonstrated (Duffy and Parmar, 2013).

### Strengths and limitations of our analysis

This study uses individual-level data for both screening and outcome data. The failure to accurately measure exposure to screening and to adopt an incidence-based mortality approach is a key limitation of studies that have examined population trends for the purpose of evaluating breast screening (Moss *et al*, 2012). Our IBM analyses were restricted to women free from breast cancer at entry to avoid dilution of the effect of screening; 57% of breast cancer deaths occurring between 1991 and 2000 were diagnosed before 1991, similar to proportions reported by others (Hakama *et al*, 1999; Duffy *et al*, 2002). The study was, however, potentially subject to a range of biases. Healthy invitee bias was minimised by using scheduling for invitation rather than invitation for screening as the measure of exposure in intention-to-screen analyses. Pro-screening lead-time bias that arises when follow-up is measured from the date of breast cancer diagnosis was avoided in our study by measuring follow-up from the start of observation. Incidence-based mortality analyses were adjusted for the form of lead-time bias that acts against screening in this type of analysis (Njor *et al*, 2012). Varying the lead-time estimate used in the adjustment to 1, 5 and 7 years resulted in estimated breast cancer mortality reductions of 17, 22 and 26%, respectively.

We were unable to ascertain dates of breast cancer diagnosis for 5% of breast cancer deaths. Sensitivity analyses excluding such cases had no impact on the estimates of the effect of screening on mortality. Findings were also unaffected when analyses were conducted excluding the 2% of deaths with unknown cause.

Findings from analyses comparing mortality in screening attenders with that in non-attenders are highly dependent on the magnitude of the correction factor used to adjust for self-selection bias. We applied a population-specific correction factor of 1.19, derived from the UK cohort study data, that was similar in magnitude to the correction factors 1.11 and 1.17 derived from Italian and Icelandic evaluation study data, respectively (Gabe *et al*, 2007; Puliti *et al*, 2008). An alternative analysis, using the correction factor of 1.36 derived from Swedish and Canadian trials (Duffy and Cuzick, 2002) resulted in a reduction of 17%. However, uptake of screening in those trials was high compared with the UK and applying a trial-derived correction to UK population screening may overcorrect (Paap *et al*, 2011). Application of a correction factor of 0.95, derived from a recent case–control evaluation of the NHSBSP (Massat *et al*, 2016) would increase estimated breast cancer mortality reduction amongst attenders for screening in our study to 50%.

There were concerns that screening exposure data collected from the breast screening call/recall system might not accurately reflect screening prior to 1995. Validation by checking detailed screening histories of over 100 000 individuals indicated a high level of accuracy and completeness overall, but there was evidence of some missing screening information in some areas before 1995. The effect of this would be to dilute a positive effect of screening.

Temporal differences between exposure groups in our intention to screen IBM analyses mean they are potentially confounded by changes in non-screening factors over time. Falling UK breast cancer mortality rates since 1990 are likely to be due to a combination of factors, including improvements in treatment and the direct effect of screening through earlier detection and treatment. In addition, there are likely to be indirect screening effects which include increased breast awareness associated with promotion of the NHSBSP (Stockton *et al*, 1997) and better access to multi-disciplinary breast care (Department of Health and Welsh Office, 1995; Kalager *et al*, 2010). Although we have identified a reduction in breast cancer mortality associated with NHSBSP screening, we were not able to differentiate the contribution made by the direct and indirect effects of screening. Temporal differences between exposure groups in our intention-to-screen IBM analyses mean they are potentially confounded by changes in non-screening factors over time. However, these temporal differences were relatively small, thus minimising the likelihood of confounding due to changes in non-screening factors. Furthermore, use of Tamoxifen and adjuvant therapy was widespread during the period covered by this evaluation (Alexander *et al*, 1994; Moritz *et al*, 1997; Swerdlow and Jones, 2005) so that changes in these factors are unlikely to have substantially affected the results.

Findings from this large evaluation of the NHSBSP are similar to those reported by cohort evaluations of organised screening in other countries (Jonsson *et al*, 2001; Paci *et al*, 2002; Olsen *et al*, 2005; Swedish Organised Service Screening Evaluation Group, 2006; Weedon-Fekjaer *et al*, 2014; Parvinen *et al*, 2015). Our analyses primarily cover NHSBSP activity up to 2000; changes since 2000 have included the introduction of two-view mammography at all screens, which has increased the sensitivity of screening (Blanks *et al*, 2005), but may also have led to an increase in recall rate and/or overdiagnosis.

## Conclusions

The wide variety of approaches that have been used to estimate the impact of population breast screening reflects the difficulty of evaluating programmes that were introduced without provision of a suitable comparison population. Cohort studies using individual-level data and observed mortality represent a robust approach to evaluation and this study is the first evaluation of the NHS breast screening programme to adopt such a strategy. This cohort study adds considerably to the body of evidence indicating that population-based mammographic screening leads to a reduction in breast cancer mortality.

## Figures and Tables

**Table 1:** Breast cancer mortality in invited compared with uninvited women

					**Rate ratio (95% CI,** ***P*****-value)**		
**Exposure status**	**Number of women**	**Number of person-years**	**Number of breast cancer deaths**	**Crude breast cancer mortality rate per 1000 person-years**	**Crude**	**Adjusted for age and socioeconomic status**^a^	**Adjusted for age, socioeconomic status and lead-time**^b^
**Incidence-based mortality**							
Not invited	988 090	1 675 356	1239	0.74	1.00	1.00	1.00
Invited		4 719 228	2912	0.62	0.83 (0.78–0.89, <0.001)	0.82 (0.76–0.88, <0.001)	0.79 (0.73–0.84, <0.001)
**Late- and early-starting screening areas**							
Late-starting	52 949	723 558	490	0.68	1.00	1.00	n/a^c^
Early-starting	49 713	685 758	373	0.54	0.80 (0.70–0.92, 0.001)	0.82 (0.71–0.94, 0.004)	n/a^c^

Abbreviation: CI=confidence interval.

a Adjusted for single year of age and socioeconomic status quintile.

b Adjusted for lead-time of 3 years.

c Lead-time does not affect this analysis.

**Table 2:** Breast cancer mortality (conventional) in attenders and non-attenders in response to first round screening invitation: women aged 49–64 years at invitation

					**Rate ratio (95% CI,** ***P*****-value)**		
**Exposure status**	**Number of women**	**Number of person-years**	**Number of breast cancer deaths**	**Rate per 1000 person-years**	**Unadjusted**	**Adjusted for age and socioeconomic status**^a^	**Adjusted for age, socioeconomic status and selection bias**^b^
Attenders	587 809	7 411 762	3120	0.42	0.54 (0.51–0.57, <0.001)	0.54 (0.51–0.57, <0.001)	0.68 (0.63–0.73, <0.001)
Non-attenders	203 137	2 347 909	1845	0.79	1.00	1·00	1.00

Abbreviation: CI=confidence interval.

a Adjusted for single year of age and socioeconomic status quintile.

b Adjusted for self-selection bias using non-attender to uninvited breast cancer mortality ratio.

## References

[bib1] Alexander FE, Anderson TJ, Brown HK, Forrest AP, Hepburn W, Kirkpatrick AE, McDonald C, Muir BB, Prescott RJ, Shepherd SM (1994) The Edinburgh randomised trial of breast cancer screening: results after 10 years of follow-up. Br J Cancer 70(3): 542–548. 8080744 10.1038/bjc.1994.342 PMC2033341

[bib2] Allgood PC, Warwick J, Warren RM, Day NE, Duffy SW (2008) A case-control study of the impact of the East Anglian breast screening programme on breast cancer mortality. Br J Cancer 98(1): 206–209. 18059396 10.1038/sj.bjc.6604123 PMC2359716

[bib3] Biesheuvel C, Barratt A, Howard K, Houssami N, Irwig L (2007) Effects of study methods and biases on estimates of invasive breast cancer overdetection with mammography screening: a systematic review. Lancet Oncol 8(12): 1129–1138. 18054882 10.1016/S1470-2045(07)70380-7

[bib4] Blanks RG, Bennett RL, Patnick J, Cush S, Davison C, Moss SM (2005) The effect of changing from one to two views at incident (subsequent) screens in the NHS breast screening programme in England: impact on cancer detection and recall rates. Clin Radiol 60(6): 674–680. 16038694 10.1016/j.crad.2005.01.008

[bib5] Blanks RG, Moss SM, McGahan CE, Quinn MJ, Babb PJ (2000) Effect of NHS breast screening programme on mortality from breast cancer in England and Wales, 1990-8: comparison of observed with predicted mortality. BMJ 321(7262): 665–669. 10987769 10.1136/bmj.321.7262.665 PMC27479

[bib6] Bleyer A, Baines C, Miller AB (2016) Impact of screening mammography on breast cancer mortality. Int J Cancer 138(8): 2003–2012. 26562826 10.1002/ijc.29925

[bib7] Broeders M, Moss S, Nystrom L, Njor S, Jonsson H, Paap E, Massat N, Duffy S, Lynge E, Paci E (2012) The impact of mammographic screening on breast cancer mortality in Europe: a review of observational studies. J Med Screen 19(Suppl 1): 14–25. 10.1258/jms.2012.012078 22972807

[bib8] Department of Health and Welsh Office (1995) A Policy Framework for Commissioning Cancer Services. Department of Health: London, UK.

[bib9] Duffy SW, Cuzick J (2002) Correcting for non-compliance bias in case-control studies to evaluate cancer screening programmes. Appl Stat 51(2): 235–243.

[bib10] Duffy SW, Parmar D (2013) Overdiagnosis in breast cancer screening: the importance of length of observation period and lead time. Breast Cancer Res 15(3): R41. 23680223 10.1186/bcr3427 PMC3706885

[bib11] Duffy SW, Tabar L, Chen HH, Holmqvist M, Yen MF, Abdsalah S, Epstein B, Frodis E, Ljungberg E, Hedborg-Melander C, Sundbom A, Tholin M, Wiege M, Akerlund A, Wu HM, Tung TS, Chiu YH, Chiu CP, Huang CC, Smith RA, Rosen M, Stenbeck M, Holmberg L (2002) The impact of organized mammography service screening on breast carcinoma mortality in seven Swedish counties. Cancer 95(3): 458–469. 12209737 10.1002/cncr.10765

[bib12] Duffy SW, Tabar L, Olsen AH, Vitak B, Allgood PC, Chen TH, Yen AM, Smith RA (2010) Absolute numbers of lives saved and overdiagnosis in breast cancer screening, from a randomized trial and from the Breast Screening Programme in England. J Med Screen 17(1): 25–30. 20356942 10.1258/jms.2009.009094 PMC3104821

[bib13] Fielder HM, Warwick J, Brook D, Gower-Thomas K, Cuzick J, Monypenny I, Duffy SW (2004) A case-control study to estimate the impact on breast cancer death of the breast screening programme in Wales. J Med Screen 11(4): 194–198. 15563774 10.1258/0969141042467304

[bib14] Gabe R, Tryggvadottir L, Sigfusson BF, Olafsdottir GH, Sigurdsson K, Duffy SW (2007) A case-control study to estimate the impact of the Icelandic population-based mammography screening program on breast cancer death. Acta Radiol 48(9): 948–955. 18080359 10.1080/02841850701501725

[bib15] Hakama M, Pukkala E, Soderman B, Day N (1999) Implementation of screening as a public health policy: issues in design and evaluation. J Med Screen 6(4): 209–216. 10693068 10.1136/jms.6.4.209

[bib16] Health & Social Care Information Centre (2016) Breast Screening Programme, England, 2014–215. Available at www hscic gov uk.

[bib17] Independent UK Panel on Breast Cancer Screening (2012) The benefits and harms of breast cancer screening: an independent review. Lancet 380(9855): 1778–1786. 23117178 10.1016/S0140-6736(12)61611-0

[bib18] Jonsson H, Nystrom L, Tornberg S, Lenner P (2001) Service screening with mammography of women aged 50-69 years in Sweden: effects on mortality from breast cancer. J Med Screen 8(3): 152–160. 11678556 10.1136/jms.8.3.152

[bib19] Jorgensen KJ, Gotzsche PC (2010) Who evaluates public health programmes? A review of the NHS Breast Screening Programme. J R Soc Med 103(1): 14–20. 20056665 10.1258/jrsm.2009.090342 PMC2802704

[bib20] Jorgensen KJ, Gotzsche PC (2016) Breast cancer: Updated screening guidelines—much ado about small improvements. Nat Rev Clin Oncol 13(3): 139–140. 26718110 10.1038/nrclinonc.2015.232

[bib21] Kalager M, Zelen M, Langmark F, Adami HO (2010) Effect of screening mammography on breast-cancer mortality in Norway. N Engl J Med 363(13): 1203–1210. 20860502 10.1056/NEJMoa1000727

[bib22] Lauby-Secretan B, Scoccianti C, Loomis D, Benbrahim-Tallaa L, Bouvard V, Bianchini F, Straif K (2015) Breast-cancer screening–-viewpoint of the IARC Working Group. N Engl J Med 372(24): 2353–2358. 26039523 10.1056/NEJMsr1504363

[bib23] Massat NJ, Dibden A, Parmar D, Cuzick J, Sasieni PD, Duffy SW (2016) Impact of Screening on Breast Cancer Mortality: The UK Program 20 Years On. Cancer Epidemiol Biomarkers Prev 25(3): 455–462. 26646362 10.1158/1055-9965.EPI-15-0803

[bib24] Michalopoulos D, Duffy SW (2016) Estimation of overdiagnosis using short-term trends and lead time estimates uncontaminated by overdiagnosed cases: Results from the Norwegian Breast Screening Programme. J Med Screen 23(4): 192–202. 26940963 10.1177/0969141315623980 PMC5098694

[bib25] Moritz S, Bates T, Henderson SM, Humphreys S, Michell MJ (1997) Variation in management of small invasive breast cancers detected on screening in the former south east Thames region: observational study. BMJ 315(7118): 1266–1272. 9390053 10.1136/bmj.315.7118.1266 PMC2127781

[bib26] Moss SM, Nystrom L, Jonsson H, Paci E, Lynge E, Njor S, Broeders M (2012) The impact of mammographic screening on breast cancer mortality in Europe: a review of trend studies. J Med Screen 19(Suppl 1): 26–32. 10.1258/jms.2012.012079 22972808

[bib27] Njor S, Nystrom L, Moss S, Paci E, Broeders M, Segnan N, Lynge E (2012) Breast cancer mortality in mammographic screening in Europe: a review of incidence-based mortality studies. J Med Screen 19(Suppl 1): 33–41. 22972809 10.1258/jms.2012.012080

[bib28] Olsen AH, Njor SH, Vejborg I, Schwartz W, Dalgaard P, Jensen MB, Tange UB, Blichert-Toft M, Rank F, Mouridsen H, Lynge E (2005) Breast cancer mortality in Copenhagen after introduction of mammography screening: cohort study. BMJ 330(7485): 220. 15649904 10.1136/bmj.38313.639236.82 PMC546064

[bib29] Paap E, Verbeek AL, Puliti D, Paci E, Broeders MJ (2011) Breast cancer screening case-control study design: impact on breast cancer mortality. Ann Oncol 22(4): 863–869. 20924073 10.1093/annonc/mdq447

[bib30] Paci E, Broeders M, Hofvind S, Puliti D, Duffy SW (2014) European breast cancer service screening outcomes: a first balance sheet of the benefits and harms. Cancer Epidemiol Biomarkers Prev 23(7): 1159–1163. 24991022 10.1158/1055-9965.EPI-13-0320

[bib31] Paci E, Duffy SW, Giorgi D, Zappa M, Crocetti E, Vezzosi V, Bianchi S, del Turco MR (2002) Quantification of the effect of mammographic screening on fatal breast cancers: The Florence Programme 1990-96. Br J Cancer 87(1): 65–69. 12085258 10.1038/sj.bjc.6600301 PMC2364283

[bib32] Parvinen I, Heinavaara S, Anttila A, Helenius H, Klemi P, Pylkkanen L (2015) Mammography screening in three Finnish residential areas: comprehensive population-based study of breast cancer incidence and incidence-based mortality 1976-2009. Br J Cancer 112(5): 918–924. 25688742 10.1038/bjc.2014.642 PMC4453946

[bib33] Phillimore P, Beattie A, Townsend P (1994) Widening inequality of health in northern England, 1981-91. BMJ 308(6937): 1125–1128. 8173452 10.1136/bmj.308.6937.1125 PMC2540140

[bib34] Puliti D, Miccinesi G, Collina N, De Lisi V, Federico M, Ferretti S, Finarelli AC, Foca F, Mangone L, Naldoni C, Petrella M, Ponti A, Segnan N, Sigona A, Zarcone M, Zorzi M, Zappa M, Paci E (2008) Effectiveness of service screening: a case-control study to assess breast cancer mortality reduction. Br J Cancer 99(3): 423–427. 18665188 10.1038/sj.bjc.6604532 PMC2527797

[bib35] Puliti D, Miccinesi G, Zappa M, Manneschi G, Crocetti E, Paci E (2012) Balancing harms and benefits of service mammography screening programs: a cohort study. Breast Cancer Res 14(1): R9. 22230345 10.1186/bcr3090 PMC3496124

[bib36] Richardson A (2001) Screening and the number needed to treat. J Med Screen 8(3): 125–127. 11678550 10.1136/jms.8.3.125

[bib37] Stockton D, Davies T, Day N, McCann J (1997) Retrospective study of reasons for improved survival in patients with breast cancer in east Anglia: earlier diagnosis or better treatment. BMJ 314(7079): 472–475. 9056796 10.1136/bmj.314.7079.472 PMC2126011

[bib38] Svendsen AL, Olsen AH, von Euler-Chelpin M, Lynge E (2006) Breast cancer incidence after the introduction of mammography screening: what should be expected? Cancer 106(9): 1883–1890. 16572411 10.1002/cncr.21823

[bib39] Swedish Organised Service Screening Evaluation Group (2006) Reduction in breast cancer mortality from organized service screening with mammography: 1. Further confirmation with extended data. Cancer Epidemiol Biomarkers Prev 15(1): 45–51. 16434585 10.1158/1055-9965.EPI-05-0349

[bib40] Swerdlow AJ, Jones ME (2005) Tamoxifen treatment for breast cancer and risk of endometrial cancer: a case-control study. J Natl Cancer Inst 97(5): 375–384. 15741574 10.1093/jnci/dji057

[bib41] Weedon-Fekjaer H, Romundstad PR, Vatten LJ (2014) Modern mammography screening and breast cancer mortality: population study. BMJ 348: g3701. 24951459 10.1136/bmj.g3701 PMC4061379

[bib42] Weedon-Fekjaer H, Vatten LJ, Aalen OO, Lindqvist B, Tretli S (2005) Estimating mean sojourn time and screening test sensitivity in breast cancer mammography screening: new results. J Med Screen 12(4): 172–178. 16417693 10.1258/096914105775220732

[bib43] Wilson RM, Evans AJ (2006) Over-diagnosis and breast cancer screening. Eur J Cancer 4(2): 6–9.

