# Supplementary material for: Effect of population breast screening on breast cancer mortality up to 2005 in England and Wales: an individual-level cohort study
Source: Br J Cancer. 2016 Dec 8;116(2):246–52. doi: 10.1038/bjc.2016.415 (PMC5243996; doi:10.1038/bjc.2016.415)
Supplement: Supplementary Information [file bjc2016415x1.docx]

**Johns LE *et al.***

**Effect of population breast screening on breast cancer mortality up to 2005 in England and Wales: an individual-level cohort study**

**APPENDIX OF SUPPLEMENTARY INFORMATION**

**Figure A: Cohort study area**


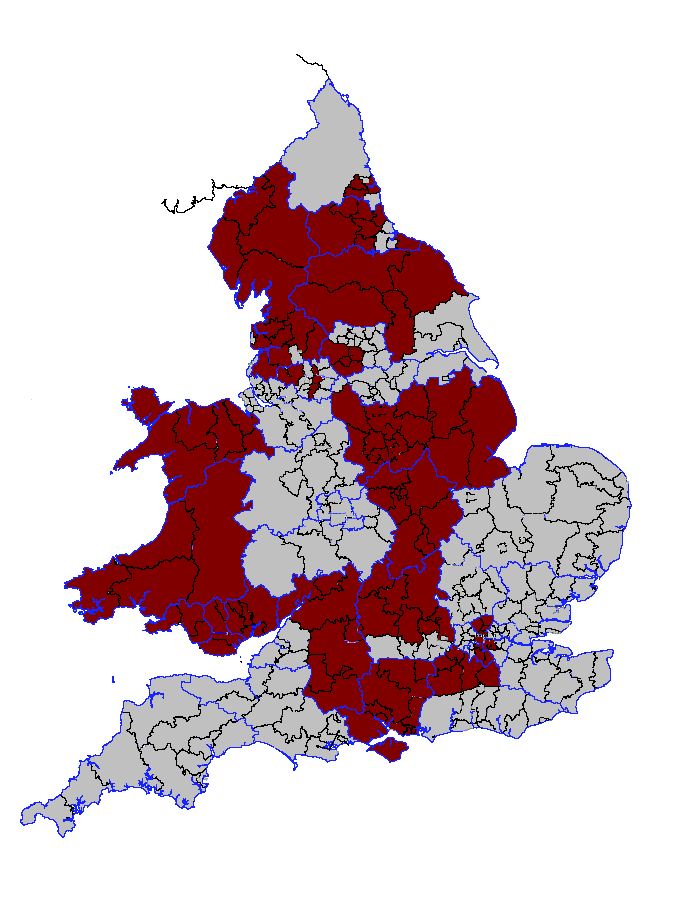


Participating areas

**1. Data sources**

Personal identification details and NHSBSP screening histories were extracted from the screening call/recall system (known as the Exeter system) in the study area. Individual personal identification data and dates of death were augmented by tracing the entire study population with the NHS Strategic Tracing Service (NSTS) (replaced by the Demographics Batch Service, part of the NHS Care Records Service). Underlying cause of death and date of breast cancer diagnosis were collected primarily from the Office for National Statistics (ONS) and in some instances from the NHS Central Registry (NHSCR, a register of all persons registered with an NHS general practitioner).

Collection of data from ONS mortality and cancer registration databases was undertaken using individual-level record linkage methods developed specifically for the study in association with ONS.

Socio-economic status was estimated using the Townsend Index (Phillimore *et al.*, 1994), from 1991 census data. Townsend metrics by enumeration district (Census Dissemination Unit, 2009) were mapped to each woman’s postcode of residence at entry or closest to her date of entry. Townsend quintiles used in analyses were defined according to the distribution of values in the whole of England and Wales.

**2. Mortality**

Breast cancer deaths were taken as those for which breast cancer was coded as the underlying cause of death, according to the International Classification of Diseases (174 under the 9th revision and C50 under the 10th revision).

**3. Breast cancer incidence**

Date of diagnosis of breast cancer was taken from the national cancer registration database. When a woman had more than one breast cancer recorded, the date of the earliest tumour was used, which could include diagnoses of *in-situ* disease. Analyses were also conducted using the date of the earliest invasive breast cancer, but this had no impact on the estimates of effect.

Any woman who had died from breast cancer, but for whom a date of diagnosis had not been ascertained through linkage with the cancer registration database, was flagged at the NHSCR to obtain cancer registration information. Following this procedure, any remaining breast cancer deaths with unknown date of diagnosis were treated as if they had been diagnosed at death.

Data on the mode of detection of breast cancer was not available from the Exeter system. Based on the distribution of the time interval between date of breast cancer diagnosis and date of last attendance for screening, cases diagnosed less than four months following a routine NHSBSP screen were categorised as screen-detected. This information was used for the purpose of adjusting for lead-time bias in incidence-based mortality analysis.

**4. Losses to follow-up**

Information from the Exeter system, the NSTS and NHSCR was used to identify losses to follow-up. Three situations led to women being classified as lost to follow-up:

1) Emigration

2) An exit date on the NSTS without indication of new location

3) Although loss of information on screening invitation and attendance was minimised by the routine transfer of screening history information when women moved from one patient register to another, screening histories were truncated when women left the study area. For the purpose of incidence-based analysis, if a woman moved out of the study area before she had been scheduled for invitation and had not exceeded the maximum age for NHSBSP invitation, she was treated as lost to follow-up since her ultimate screening exposure status could not be reliably determined.

Losses to follow-up were censored at the date of initial loss, even if there were data to show that they had subsequently died or developed breast cancer. This was done to minimise misclassification of exposure and, in the case of emigration, to avoid ‘salmon bias’, in which emigrants who become seriously ill often return to their home country (Kristensen and Bjerkedal, 2010).

**5. Impact of data limitations on the study design**

Due to the high financial cost of collecting outcome data for this cohort using conventional means (via the ONS long-term follow-up service, or ‘flagging’), alternative methodology, involving linkage of the cohort with national mortality and cancer incidence databases, was developed in close association with ONS. The quality of data collected using this methodology was assessed by means of SMRs and verification conducted in collaboration with the NHS central register. While the verification process indicated complete ascertainment of fact and date of death in the cohort, information on the underlying cause of death was incomplete for the early years of the NHSBSP, both for all causes and breast cancer deaths.

The study had been designed to evaluate NHSBSP activity from its inception in 1988, but ascertainment of cause of death was inadequate for the years 1988 and 1990. Consequently, the start date of the study was moved from 1st January 1988 to 1st January 1991.

Revision of the start date from 1st January 1988 to 1st January 1991 involved application of additional exclusion criteria; specifically, any woman who had died before 1991; been diagnosed with breast cancer before 1991; reached age 64 before 1991; been scheduled for NHSBSP invitation before 1991; or had been lost to follow-up before 1991, was excluded from the analysis. Person-years accrued prior to 1991 were not included in analyses.

**6. ‘Scheduling’ for invitation and healthy invitee bias**

In order to estimate the impact of the NHSBSP on an intention to screen basis we distinguished between women who were scheduled for invitation and those who were actually invited. We defined ‘scheduled’ as the stage when a woman’s eligibility for NHSBSP invitation has been identified on the basis of demographic information held on the Exeter system. Once identified as eligible, a routine screening episode was created by the system and it was the presence of this episode that was used to identify women scheduled for invitation. An invitation to screening was not generated until the demographic information and suitability for screening on the basis of health status had been confirmed by the relevant GP practice. Although this process of checking with the GP practice has now ceased, it was in operation during the period covered by this cohort study. This means that ill-health could result in a scheduled woman not being invited at that episode, and use of invitation as the measure of exposure in an intention to screen analysis could therefore have led to an estimate that was biased in favour of screening.

**7. Incidence-based mortality analysis**

In analyses where women could change exposure group (not scheduled for invitation or scheduled for invitation) over time, women diagnosed with breast cancer before being scheduled for invitation could die from breast cancer after being scheduled for invitation. An incidence-based mortality (IBM) approach was used in which deaths from breast cancer were assigned to an exposure group based on the woman’s exposure status at diagnosis. IBM has been employed by a number of previous screening evaluations studies, but is not always used in the same way. In our IBM analyses, women with breast cancer had their person-years censored at diagnosis and the case was counted as a breast cancer death if the woman subsequently died from breast cancer within a specified period (‘IBM follow-up’). In this way, the death was categorised as if it occurred at diagnosis.

It is important to ensure that length of IBM follow-up is similar between exposure groups. For each exposure group, there was a period to accrue breast cancer cases (‘accrual period’) and a period to encompass IBM follow-up that started at entry to the group (‘observation period’). Since most women were scheduled for invitation soon after the start of the study, the average time spent in the exposed group was greater than in the unexposed group. Without restriction, the mean length of IBM follow-up will be shorter in the exposed than the unexposed group, introducing a potential bias in favour of screening.

The analysis partitioned the 15 years available (1st January 1991 to 31st December 2005) such that the accrual period and observation period were two and nine years respectively for each exposure group. At entry, a woman was in the unexposed group and was censored after four years (by which time 98% of women had been scheduled and the average time unexposed was two years) unless scheduled or diagnosed with breast cancer. If scheduled, she entered the exposed group and accrual in this group was censored after two years.

In its simplest form, there would be one observation period for each exposure group, but the method was developed to make maximum use of available data to incorporate up to three observation periods in the exposed group, resulting in a total potential accrual of six years, extending over two three-year NHSBSP screening rounds. The observation period remained constant at nine years for every two-year accrual period. Person-years were the total person-years for all women in all accrual periods in each exposure group.

**8. Adjustment for lead-time bias**

By measuring the observation period from the start of exposure, our analyses avoided the pro-screening form of lead-time bias that arises when follow-up is measured from date of diagnosis. However, IBM introduces another effect of lead-time that inflicts a bias against screening (Njor *et al.*, 2012). Screen-detected cancers diagnosed during accrual that die after the end of accrual and within IBM-follow-up, are counted as breast cancer deaths. Some of these cancers would not have arisen during the accrual period in the absence of screening. Adjustment for this form of lead-time bias was performed by excluding screen-detected cancers diagnosed within three years of the end of each woman’s final accrual period. The period of three years was chosen from estimates of lead-time (Duffy *et al.*, 1997;Weedon-Fekjaer *et al.*, 2005;Svendsen *et al.*, 2006); further analyses were conducted using lead-time estimates of one, five and seven years.

**9. Adjustment for self-selection bias**

Analyses comparing attenders (compliers) with non-attenders (non-compliers) have been adjusted for self-selection bias using methodology developed by Duffy and Cuzick to estimate the effect of offering screening to those who would participate if invited (RR_2_) (Duffy and Cuzick, 2002). The rate ratio in attenders compared to non-attenders is adjusted to take account of the increased mortality risk in non-compliers:

P . RR_A_ . RR_N_

RR_2_

=

1 – (1 - P) . RR_N_

where, RR_A_ = r_A_/ r_N_, RR_N_ = r_N_ / r_U,_ r_A_ = breast cancer mortality rate in attenders, r_N_ = breast cancer mortality rate in non-attenders, r_U_ = breast cancer mortality rate in uninvited women. P, the proportion attending in response to first invitation was 0·74. Data from the cohort study were used to estimate r_U_ and r_N_ to derive a population-specific correction factor. The rate in uninvited women was calculated by means of conventional, non-IBM analysis.

Confidence intervals for bias-adjusted rate ratios were calculated according to the method of Cuzick *et al* (Cuzick *et al.*, 1997).

**10. Analysis of over-diagnosis**

The cumulative incidence method was used to estimate over-diagnosis, in which a comparison is made of the cumulative number of breast cancers diagnosed in the exposed group with the cumulative incidence in the unexposed group over the same time period. The comparison should be carried out several years (at least five to ten) after screening ends, in order to take into account the lead time attributable to screening (Independent UK Panel on Breast Cancer Screening, 2012). Our cumulative incidence analysis of invasive and *in situ* breast cancers was restricted to the earliest birth years in the cohort: women aged 62–64 years at entry (1st January 1991). Those that were invited to screening would have no further invitations above the age of 64 (they would have been aged over 70 in 2002 when the NHSBSP began to invite women up to age 70). There were therefore at least 12 years of follow-up for lead-time to dissipate and any over-diagnosis in the exposed group to become apparent.

Exposure on the basis of intention to screen was investigated with diagnosis of breast cancer as the outcome measure. Cumulative breast cancer incidence over the period 1st January 1991 to 31st December 2005 was compared between exposure groups. Percent over-diagnosis can be estimated a number of ways; we used methods A and B recommended by The Independent UK Panel on Breast Cancer Screening (Independent UK Panel on Breast Cancer Screening, 2012), where method A = excess breast cancers as a proportion of cases diagnosed over the whole follow-up period in uninvited women and method B = excess breast cancers as a proportion of cases diagnosed over the whole follow-up period in invited women.

**11. Mortality bias between exposure groups**

Non-breast cancer (NBC) mortality was examined for bias (*e.g*. pro-screening ‘slippery-linkage’ bias (Olsen and Gotzsche, 2001), selection bias inadequately controlled for by adjustment for socio-economic status, ascertainment bias) between exposure groups in intention to screen analysis. NBC rates in the unexposed group were higher than in the exposed group, this is likely to be due to increasing life-expectancy over time that stemmed from temporal differences between the exposure groups. Consequently, standardised mortality rates (SMRs) adjusted by single year of age and single calendar year, were used to assess non-breast cancer mortality bias between exposure groups.

**12. Number needed to schedule for invitation to save one breast cancer death**

The number needed to schedule (NNSched) was calculated from the reciprocal of the number of lives saved per woman scheduled for screening:

1 / [ ( E - O ) / N ]

where, O = observed breast cancer deaths (IBM) in scheduled women

E = expected breast cancer deaths calculated by multiplying the person-years in the scheduled group by the crude IBM rate in the unscheduled group

N = number of women in the scheduled group.

**13. Number needed to be screened to save one breast cancer death**

The number needed to be screened (NNbS) was calculated using methodology developed by Richardson (Richardson, 2001) as:

NNbS = P_a_ x NNSched

Where, P_a_ is the participation rate adjusted for selection bias (0·71) calculated as:

r_U_ – (1 – P) . r_N_

P_a_

=

r_U_

References

Census Dissemination Unit (2009) Deprivation Scores Based on 1991 Census Area Statistics. The Townsend Index. [*http://cdu*](http://cdu) *mimas ac uk/related/deprivation htm*

Cuzick J, Edwards R, Segnan N (1997) Adjusting for non-compliance and contamination in randomized clinical trials. *Stat Med* **16** (9): 1017-1029

Duffy SW, Cuzick J (2002) Correcting for non-compliance bias in case-control studies to evaluate cancer screening programmes. *Applied Statistics* **51** (2): 235-243

Duffy SW, Day NE, Tabar L, Chen HH, Smith TC (1997) Markov models of breast tumor progression: some age-specific results. *J Natl Cancer Inst Monogr* (22): 93-97

Independent UK Panel on Breast Cancer Screening (2012) The benefits and harms of breast cancer screening: an independent review. *Lancet* **380** (9855): 1778-1786, doi:S0140-6736(12)61611-0 [pii];10.1016/S0140-6736(12)61611-0 [doi]

Kristensen P, Bjerkedal T (2010) Dealing with emigration in cohort studies: follow-up of mortality and cancer incidence among Norwegians born between 1967 and 1976. *Eur J Epidemiol* **25** (3): 155-161, doi:10.1007/s10654-009-9417-9 [doi]

Njor S, Nystrom L, Moss S, Paci E, Broeders M, Segnan N, Lynge E (2012) Breast cancer mortality in mammographic screening in Europe: a review of incidence-based mortality studies. *J Med Screen* **19 Suppl 1** 33-41, doi:19/suppl_1/33 [pii];10.1258/jms.2012.012080 [doi]

Olsen O, Gotzsche PC (2001) Cochrane review on screening for breast cancer with mammography. *The Lancet* **358** (9290): 1340-1342

Phillimore P, Beattie A, Townsend P (1994) Widening inequality of health in northern England, 1981-91. *BMJ* **308** (6937): 1125-1128

Richardson A (2001) Screening and the number needed to treat. *J Med Screen* **8** (3): 125-127

Svendsen AL, Olsen AH, von Euler-Chelpin M, Lynge E (2006) Breast cancer incidence after the introduction of mammography screening: what should be expected? *Cancer* **106** (9): 1883-1890, doi:10.1002/cncr.21823 [doi]

Weedon-Fekjaer H, Vatten LJ, Aalen OO, Lindqvist B, Tretli S (2005) Estimating mean sojourn time and screening test sensitivity in breast cancer mammography screening: new results. *J Med Screen* **12** (4): 172-178, doi:10.1258/096914105775220732 [doi]
